# Safety of ultrasound-guided central venous access in critically ill patients with uncorrected coagulopathy

**DOI:** 10.1186/cc12111

**Published:** 2013-03-19

**Authors:** G Reusz, C Langer, G Egervari, P Sarkany, A Csomos

**Affiliations:** 1 Markhot Ferenc Hospital, Eger, Hungary; 2 Semmelweis University, Budapest, Hungary

## Introduction

Correction of coagulopathy before central venous catheter (CVC) insertion is a common practice; however, when ultrasound guidance is used this is controversial as mechanical complications are rare. Studies in oncology patients suggest that CVC placement without prior correction of coagulopathy is safe but no studies are available for critically ill patients and guidelines do not give recommendations [1,2]. We do not routinely correct coagulopathy, even if severe, when ultrasound guidance is used and the purpose of this retrospective study was to evaluate the safety of this practice.

## Methods

Data for all ultrasound-guided interventions, including complications, are prospectively collected in our department for audit purposes; in this study we involved only CVC insertions in the ICU between February 2011 and November 2012. Electronic medical and laboratory records and paper-based nursing charts were retrospectively studied for all interventions, specifically looking for blood results, coagulation abnormalities and intervention-related complications.

## Results

In the study period, ultrasound guidance was employed for a total of 291 central line insertions in 220 ICU patients. Coagulopathy was detected in 127 cases at the time of CVC placement (43.6%). On the day of CVC insertion, coagulation abnormalities were corrected in 20 cases (15.7%); 33 out of 50 patients with severe coagulopathy (66.0%) and 74 out of 77 patients with coagulopathy of moderate severity (96.1%) had no correction at all. Correction was started only after CVC insertion for reasons unrelated to CVC placement in a further eight and two patients with severe and less severe coagulopathy (16.0% and 2.6%), respectively. No bleeding complications were observed.

## Conclusion

In patients undergoing CVC insertion in our ICU, coagulopathy is common. We observed uncomplicated CVC placement in all 41 patients with severe uncorrected coagulopathy and in a further 76 patients with coagulopathy of moderate severity. When combined with other studies, our data suggest that ultrasound-guided CVC placement without routine correction of coagulation abnormalities may be safe in the ICU.
